# Comparison of patients receiving salicylate before coronary artery surgery with patients not receiving in terms of postoperative mortality rates

**DOI:** 10.1186/1749-8090-8-S1-P108

**Published:** 2013-09-11

**Authors:** B Ozcem, U Yetkin, M Bademci, M Akyuz, S Yazman, E Celik, N Karakas, I Yurekli, A Gurbuz

**Affiliations:** 1 Department of Cardiovascular Surgery, Izmir Katip Celebi University Ataturk Training and Research Hospital, Izmir, Turkey

## Background

The aim of this study is to detect whether there is a significant difference between patients receiving 100 mg enteric coated salicylate before coronary artery surgery with patients not receiving in terms of postoperative mortality rates.

## Methods

Sixty one patients that underwent coronary bypass surgery between January 2011 and December 2011 at our clinic were investigated retrospectively. Sixty one (80.3%) of them were operated on under cardiopulmonary bypass and 12 (19.7%) were operated on beating heart. Thirty (49.2%) of them were receivers of 100 mg enteric coated salicylate and 31 (50.8%) were non-receivers, divided into 2 groups. The mean age of salicylate receivers was 61.33 years whereas it was 57.71 years in the non-receivers.

## Results

The mortality rate was 4.5% for cases receiving salicylate that underwent coronary surgery using cardiopulmonary bypass (CPB), and 3.7% for cases not receiving salicylate. No significant difference was seen (p>0.05). No mortality was seen among patients that underwent coronary surgery on beating heart.

## Conclusions

In this study, we investigated the patient groups that underwent surgery in either method. Salicylate use did not cause any increase in mortality rate with no significant difference.

